# Flos puerariae ameliorates the intestinal inflammation of *Drosophila via* modulating the Nrf2/Keap1, JAK-STAT and Wnt signaling

**DOI:** 10.3389/fphar.2022.893758

**Published:** 2022-08-17

**Authors:** Shipei Yang, Xu Li, Minghui Xiu, Yuting Dai, Shengfang Wan, Yan Shi, Yongqi Liu, Jianzheng He

**Affiliations:** ^1^ Provincial-Level Key Laboratory for Molecular Medicine of Major Diseases and The Prevention and Treatment with Traditional Chinese Medicine Research in Gansu Colleges and University, Gansu University of Chinese Medicine, Lanzhou, China; ^2^ College of Basic Medicine, Gansu University of Chinese Medicine, Lanzhou, China; ^3^ College of Public Health, Gansu University of Chinese Medicine, Lanzhou, China; ^4^ Research Center of Traditional Chinese Medicine in Gansu, Gansu University of Chinese Medicine, Lanzhou, China; ^5^ Key Laboratory for Transfer of Dunhuang Medicine at the Provincial and Ministerial Level, Gansu University of Chinese Medicine, Lanzhou, China

**Keywords:** Flos Puerariae, intestinal inflammation, intestinal homeostasis, *Drosophila melanogaster*, intestinal stem cells, ROS

## Abstract

Gut homeostasis is important for human health, and its disruption can lead to inflammatory bowel disease (IBD). Flos Puerariae is a herb with a wide variety of pharmacological activities including antioxidant, antidiabetic, antialcoholismic and anti-inflammatory properties. However, the role of Flos Puerariae on treating IBD remains obscure. Here, we employed *Drosophila melanogaster* as a model organism to investigate the protective effect of Flos Puerariae extract (FPE) against sodium dodecyl sulfate (SDS)-induced intestinal injury. Our data showed that FPE had no toxic effect in flies, and significantly extended lifespan in SDS-inflamed flies, reduced stem cell proliferation in the midgut, and maintained intestinal morphological integrity. Furthermore, FPE remarkably recused the altered expression level of genes and proteins in Nrf2/Keap1 signaling, JAK-STAT signaling and Wnt signaling pathways in gut of inflammation flies. Thus, FPE has a protective effect against intestinal injury possibly via increasing the Nrf2/keap1 pathway and suppressing the JAK-STAT and Wnt signaling pathways, which would have tremendous potential for treating IBD.

## Introduction

Inflammatory bowel disease (IBD) is a blanket term that refers to a set of immunological disorders affecting the gastrointestinal system that has no recognized cause (Mentella et al., 2020). There are several clinical manifestations in individuals with IBD, including diarrhea and bloody stool, and the majority of them also have extra intestinal complications such as arthritis, dermatitis, and vision loss (Anbazhagan et al., 2018; Hedin et al., 2019). There are no particular treatments of IBD at the present time, and practically all medications just reduce symptoms. Due to the high risk of recurrence rate and the high expense of therapy, it has a negative impact on patients’ quality of life and adds to their emotional and financial burden (Abraham et al., 2017; Neurath, 2017; Xie et al., 2021). As a result, it is critical to develop new natural products that are both effective and economical for treating IBD.

The etiology of IBD is yet unknown, although its pathophysiology is though to include more than 100 genetic and environmental variables, including smoking, diet, and stress (Mak et al., 2020). Epithelial homeostasis is normally maintained *via* a balance of intestinal stem cell (ISC) self-renewal, progenitor differentiation, cell shedding and apoptosis (Hou et al., 2021). ISCs proliferation is activated as the intestinal morphology damage to regenerate the damage intestinal epithelium (Wu et al., 2020). Several signaling pathways have been reported to regulate ISCs proliferation and intestinal homeostasis, such as JAK-STAT pathway, Wnt signaling pathway and mTOR signaling pathway (Barthez et al., 2020; Böttcher et al., 2021; Moon et al., 2021). In inflammatory states, JAK-STAT pathway is activated to promote the proliferation and differentiation of ISCs (Moon et al., 2021). Also, extensive ROS induces oxidative stress, resulting in intestinal disorder and ISCs over-proliferation (Wisidagama and Thummel, 2019; Zhang et al., 2020). Activation of the Nrf2/keap1 signaling pathway could provide an endogenous defence system against cellular oxidative stress and attenuate oxidative damage, making it a promising therapeutic mechanism for the treatment of IBD (Jaiswal, 2004; Maloy and Powrie, 2011). *Drosophila melanogaster* is a well-established model organism for elucidating the mechanisms behind intestinal inflammation. D*rosophila* and human are comparable in morphology and physiological function, as well as in cell composition and the signaling pathways that drive intestinal regeneration (Arbouzova & Zeidler, 2006; He et al., 2022). The JAK-STAT, Wnt signaling and Nrf2/Keap1 pathways are evolutionarily conserved from *Drosophila* to humans, and are involved in gut homeostasis and tissue regeneration (Fuller & Spradling, 2007; Pitoniak & Bohmann, 2015; Ren et al., 2015). As a result, *Drosophila* is a very dependable model for the study of intestinal disorders. Recently, more and more studies are focused on how different herb influence ISC function and epithelial homeostasis.

The blossoms of *Pueraria lobata (Wild.)* are known as *‘*Flos Puerariae’. It is a perennial herbaceous plant of the Fabaceae family, that is, found across temperate regions of China, Korea, Japan, and India (Lertpatipanpong et al., 2020). Flos Puerariae as one of the widely used medicinal herbs exhibits a variety of biological actions, including anti-inflammatory, antioxidant, hypoglycemic and antidiabetic properties (Eom et al., 2018). It is edible and is also processed into many dishes (Tousen et al., 2019). In China, medications made from Flos Puerariae have been found to alleviate fever, reduce stiffness and discomfort, and function as an antiphlogistic (Zhang et al., 2012b). Modern pharmacological studies have demonstrated that Flos Puerariae could alleviate ovariectomized-induced osteoporosis in rats and inhibit growth of breast and ovarian cancer cells (Satpathy et al., 2021). Genistein could block the JAK-STAT signaling pathway through inhibiting the expressions of STAT1, STAT3 and STAT6 *in vitro* and *in vivo* (Hämäläinen et al., 2007; Gao et al., 2012; Wang et al., 2019a). Daidzein is also able to combat inflammatory stress and JAK-STAT signaling pathway to protect dopamine neurons *in vitro* inflammation models (Hämäläinen et al., 2007). However, the therapeutic benefits and mechanisms of Flos Puerariae extract (FPE) on inflammatory bowel disease under stressful conditions are not clear.

In this study, we explored the protective effect of FPE against intestinal injury induced by SDS in *Drosophila* flies. It was demonstrated that FPE could extend the lifespan of flies under inflammatory setting, decrease epithelial cell damage, morphological changes, and ISCs over-proliferation in the gut, and rescue the SDS- induced alternation in Nrf2/keap1 signaling, JAK-STAT signaling and Wnt signaling pathways. Thus, FPE may protect the intestinal tract under stress conditions and thus has significant therapeutic utility in IBD.

## Materials and methods

### *Drosophila* strain and maintenance

All flies were reared on standard medium at 25 C and approximately 65% humidity under a 12 h light/12 h dark cycle. Flies lines used in this study were as follows: *esg-Gal4*, UAS-*GFP* was kindly provided by Dr. Lihua Jin, Northeast Forestry University; *10×STAT92E-GFP* and *gstD1-GFP* lines were gifts from Dr Fengwei Yu, Temasek Life Sciences Laboratory, National University of Singapore. *w* ^*1118*^ (#5905) were obtained from the Bloomington *Drosophila* stock center (BDSC; Indiana, United States). 3–5 day old adult female or male were collected using light CO_2_ anesthesia and allowed to recover for 2 days before further experimentation.

### Drug treatment

Flos Puerariae extract (FPE) was purchased from Shaanxi Ruilin parnell biotechnology Co. Ltd. The dried Flos Puerariae was mixed with 50% (V/V) methanol solution with the solid-to-liquid ratio at 1∶30. The mixture was extracted with ultrasound for 2 h at 70°C. The extracted products were then purified sequentially by petroleum ether, ethanol and chloroform-butyl alcohol, and eluted gradually with mixed mobile phase of methanol-chloroform solution in the silica gel column system. In the end, the isolated ingredients were further analyzed by color reaction, ultraviolet spectrophotometry, high performance liquid chromatography, infrared spectrum and mass spectrum. FPE was diluted with a standard cornmeal-molasses medium to different concentrations (0, 1, 5 and 10 mg/ml). *w* ^*1118*^ flies were supplied with different concentrations of FPE in their standard diet from the 1st instar larvae until experiments. 0.4 mm ML385 (SML 1,833, Sigma, China) and 0.6% SDS together were provided to adult females during experiments.

### Quantitative analysis

Liquid chromatography coupled to mass spectrometry (LC-MS) was used as a reference standard for the quality control of FPE. LC-MS analysis was performed as previously described (Wang et al., 2020). Separation was achieved by utilizing a Ascentis Express C18 (100 mm × 2.1 mm i.d., 1.8 µM). Formic acid (0.05%, V/V) with water (eluent A) and acetonitrile (eluent B) were used for gradient elution with the following time program (%B in A): from 0 to 20 (1 min), from 20 to 70 (8 min), from 70 to 95 (3.5 min), from 95 to 0 (1 min), and finally isocratic reconditioning for 1 min. The flow rate was set at 0.35 ml/min and the temperature was set at 40°C. The injection volume is 2 μL. Data acquisitions were performed using Analyst 1.6.3 software (Sciex). Multiquant 3.0.3 software (Sciex) was used to quantify all metabolites.

### Survival rate

3–5 days old males or females of the 4 groups (including the control groups and the experimental groups) fed the corresponding mediums were cultured for 2 days, and then removed to empty vials for 2 h. 20 flies per vial were transferred into vials containing five layers of filter paper infiltrated by 0.6% SDS and 5% sucrose solution, or 5% H_2_O_2_ and 5% sucrose solution, respectively. Filter papers were replaced every 2 days and the number of dead flies was recorded. Four treatments were performed, and each with at least three replicate vials.

### “Smurfs” assay

The “Smurfs” assay, as previously described, refers primarily to the use of a non-absorbent blue dye (FD&C Blue #1) to analyse the intestinal barrier integrity of *Drosophila* (Rera et al., 2011). Briefly, after fed in the medium containing 5% sucrose with or without 0.6% SDS for 72 h, females were transferred to the medium with blue dye (2.5% w/v) for 18 h. The number of “Smurfs” was then calculated.

### Sodium dodecyl sulfate feeding assay

3–5 days old females were starved in empty vials for 2 h at 25°C, then 20 females per vial were transferred to vials containing 5 layers of filter paper on the bottom. Filter papers were wetted with 5% sucrose solution (control) or 0.6% SDS and 5% sucrose solution (experiment). After 16 h, guts were dissected and examined.

### Immunostaining

After fed in the medium containing 5% sucrose with or without 0.6% SDS for 16 h, 15–20 females per group were used to dissect intestines in cold PBS, the isolated guts were fixed with 3.7% formaldehyde for 30 min at room temperature. Following three washes with PBST for 10 min each, the samples were stained with 4’ 6-diamidino-2-phenylindole (DAPI) for 10 min. The tissues were then mounted in cedar wood oil (Beijing Solarbio Science & Technology Co., Ltd, China) and observed under an Olympus FV1000 confocal microscope (Olympus, Japan). ISCs were identified by their esg-GFP expression, their size, and their basal location within the intestinal epithelium. The experiments were independently repeated at least three times.

### Quantitative RT-PCR analysis

Adult females were incubated with 0.6% SDS and 5% sucrose medium or individual 5% sucrose for 16 h, then total RNA of 60 females was extracted with TRIzol reagent (Invitrogen, Carlsbad, CA, UAS) according to manufacturer’s instructions. Total RNA was reverse transcribed using Hieff® reverse transcriptase (Shanghai YEASEN, China). Quantitative PCR was performed with a GFX 96 Connect^TM^ Optics Module (Bio-Rad Laboratories) using Multiplex PCR Master Mix (Shanghai YEASEN, China). Ribosomal gene *RP49* was used as the internal control. The primer sequences were listed in Table 1. The gene expression was calculated using the comparative threshold cycle (Ct) method. The levels of gene expression in all groups were shown as a ratio to the SDS treated control group value. At least three replicates were established for each group, and all experiments were repeated at least three times.

**TABLE 1:** List of forward and reverse primers used in gene expression studies.

Genes	Forward	Reverse
*Sod1*	GCGGCGTTATTGGCATTG	ACT​AAC​AGA​CCA​CAG​GCT​ATG
*Sod2*	CAC​ATC​AAC​CAC​ACC​ATC​TTC	GCTCTTCCACTGCGACTC
*Cat*	TGA​ACT​TCC​TGG​ATG​AGA​TGT​C	TCT​TGG​CGG​CAC​AAT​ACT​G
*gstD1*	TGA​TCA​ATC​AGC​GCC​TGT​ACT	GCA​ATG​TCG​GCT​ACG​GTA​AG
*gstD2*	CGGACATTGCCATCCTGT	TGC​TGA​AGT​CGA​ACT​CAC​TAA​CTT
*dKeap1*	CAA​GGA​GTC​GGA​GAT​GTC​G	GTA​GAG​GAT​GCG​TGA​CAT​GG
*CncC*	GAG​GTG​GAA​ATC​GGA​GAT​GA	CTG​CTT​GTA​GAG​CAC​CTC​AGC
*Upd*	CCA​CGT​AAG​TTT​GCA​TGT​TG	CTA​AAC​AGT​AGC​CAG​GAC​TC
*Upd2*	CGG​AAC​ATC​ACG​ATG​AGC​GAA​T	TCG​GCA​GGA​ACT​TGT​ACT​CG
*Upd3*	CCC​AGC​CAA​CGA​TTT​TTA​TG	TGTTACCGCTCCGGCTAC
*Hop*	CTGGGCTCCAAGATACG	GGCAGATACTGAACGGTG
*Socs36E*	CAG​TCA​GCA​ATA​TGT​TGT​CG	ACT​TGC​AGC​ATC​GTC​GCT​TC
*Wg*	*GAT​TAT​TCC​GCA​GTC​TGG​TC*	*CTA​TTA​TGC​TTG​CGT​CCC​TG*
*Arm*	*TTC​GGA​ACC​GTC​ACA​AAT​GC*	*ATC​CTC​ATC​GTT​CAG​CAG​CC*
*Rp49*	CTTCATCCGCCACCAGTC	GCA​CCA​GGA​ACT​TCT​TGA​ATC

### Statistical analysis

Statistical analysis was performed using GraphPad Prism software. Comparisons among groups were made by analysis of variance (ANOVA) followed by Dunnett’s t-test. Survivorships among groups were compared and tested for significance with a Log-rank test. Differences between treatment groups were considered to be statistically significant at *p < 0.05, **p < 0.01, ***p < 0.001 and ****p < 0.0001.

## Results

### Identification of bioactive compounds in Flos Puerariae extract using LC-MS

**FIGURE 1:**
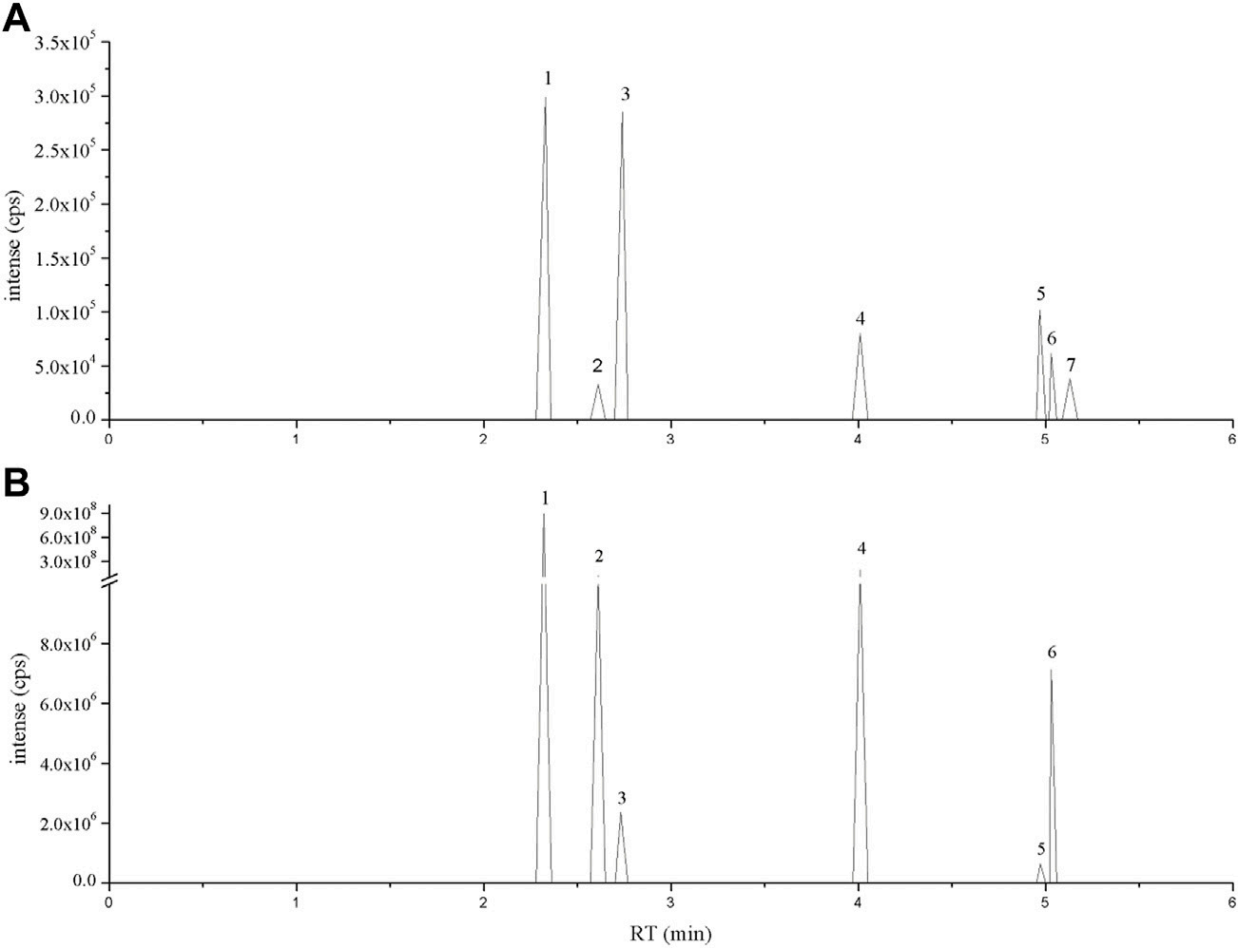
LC-MS ion-flow chromatogram of **(A)** Standard and **(B)** FPE. (1) Puerarin, (2) Daidzin, (3) Rutin, (4) Daidzein, (5) Apigenin, (6) Genistein and (7) Tectorigenin.

The primary constituent of Flos Puerariae is isoflavonoids, including puerarin, daidzein, daidzin, genistein and apigenin (Eom et al., 2018; Lertpatipanpong et al., 2020). To identify these bioactive compounds in FPE, LC-MS analysis was used. The chromatogram of the standard was eluted at a retention time of 30 min, and it was determined that FPE contained puerarin, daidzein, daidzin, rutin, apigenin and genistein (Figure 1).

### Flos puerariae extract increases the survival rate following the ingestion of sodium dodecyl sulfate

**FIGURE 2:**
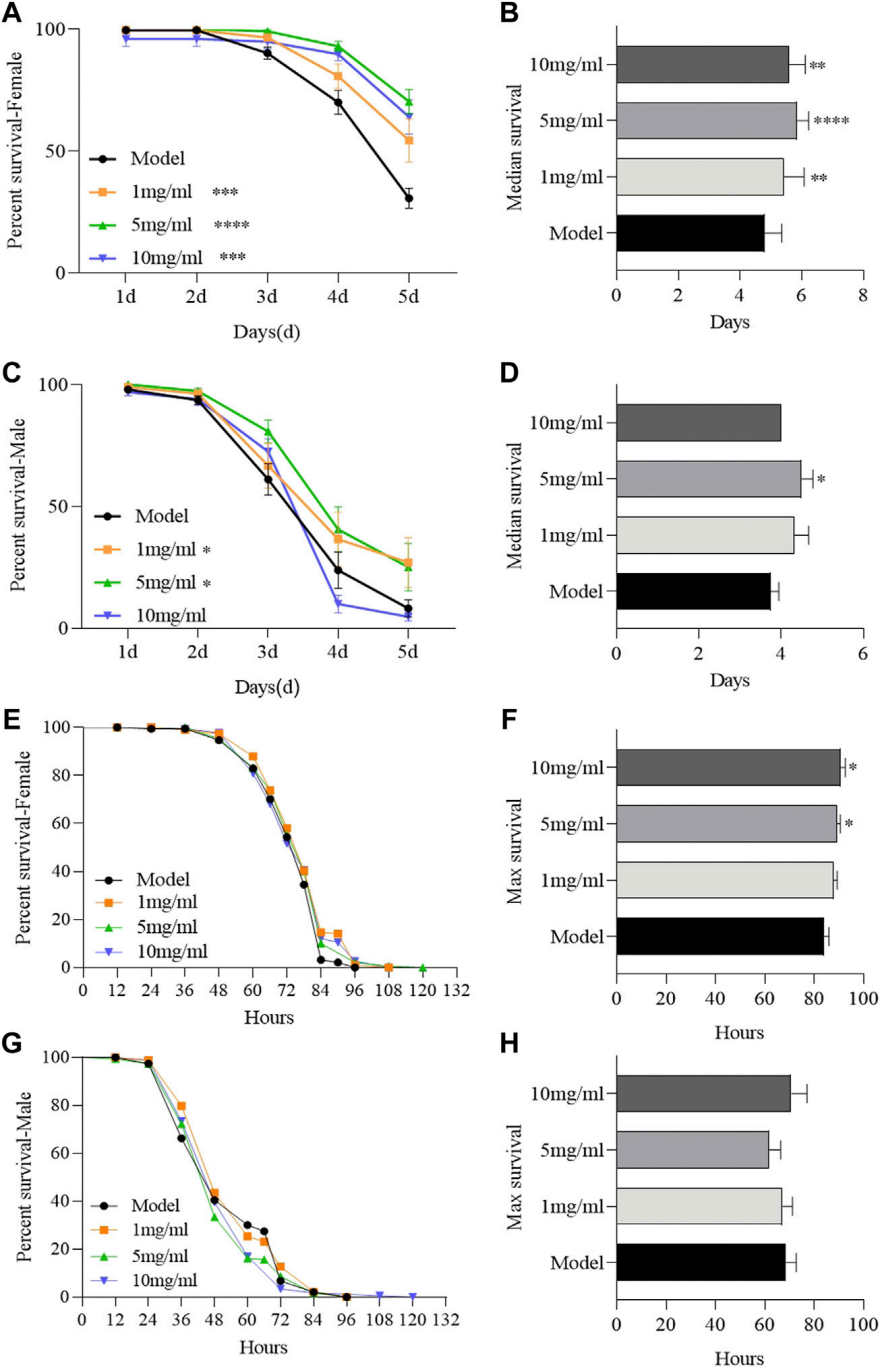
FPE ameliorates survival rates of flies during treated with SDS or H_2_O_2_. *w* ^*1118*^ flies were reared with standard foods (control group), or with foods supplemented with FPE (1, 5 and 10 mg/ml, respectively). Survivorship curves in different groups of females **(A)** and males **(C)** fed the food with 0.6% SDS. The median lifespan in females **(B)** and males **(D)** fed the food with 0.6% SDS. Survivorship curves in different groups of females **(E)** and males **(G)** fed the food with 5% H_2_O_2_. The maximal lifespan in females **(F)** and males **(H)** fed the food with 5% H_2_O_2_. Log-rank *p* values between survival curves are shown. The results are presented as the means ± SEM (*n* = 10–16). *p < 0 0.05, **p < 0.01, ***p < 0.001, ****p < 0.0001.

To determine the protective effect of FPE, adult females or males were orally treated with inflammatory reagent or ROS-producing agents, SDS or H_2_O_2_ respectively. SDS interferes with the normal function of the intestinal barrier and stimulates local and systemic inflammation (Song et al., 2021). Survival rates were remarkably increased in FPE treated females or males compared to the control flies, following exposure to SDS (Figures 2A–D). FPE had stronger protective function in females than in males, in which 10 mg/ml FPE treated males did not enhance the survival rates under SDS stimulation. FPE treated flies did not exhibit significantly extended survival rates compared to the control flies under H_2_O_2_ stimulation (Figures 2E,G). However, females fed with 5 mg/ml and 10 mg/ml FPE remarkably increased the maximal life span (Figures 2F,H), indicating FPE had weak antioxidant ability *in vivo*. Thus, these findings indicate that FPE has protection against chemical-induced inflammatory injury in flies, and the protective function of FPE is stronger in females than in males.

### Flos puerariae extract has no function in regulating development and food consumption

**FIGURE 3:**
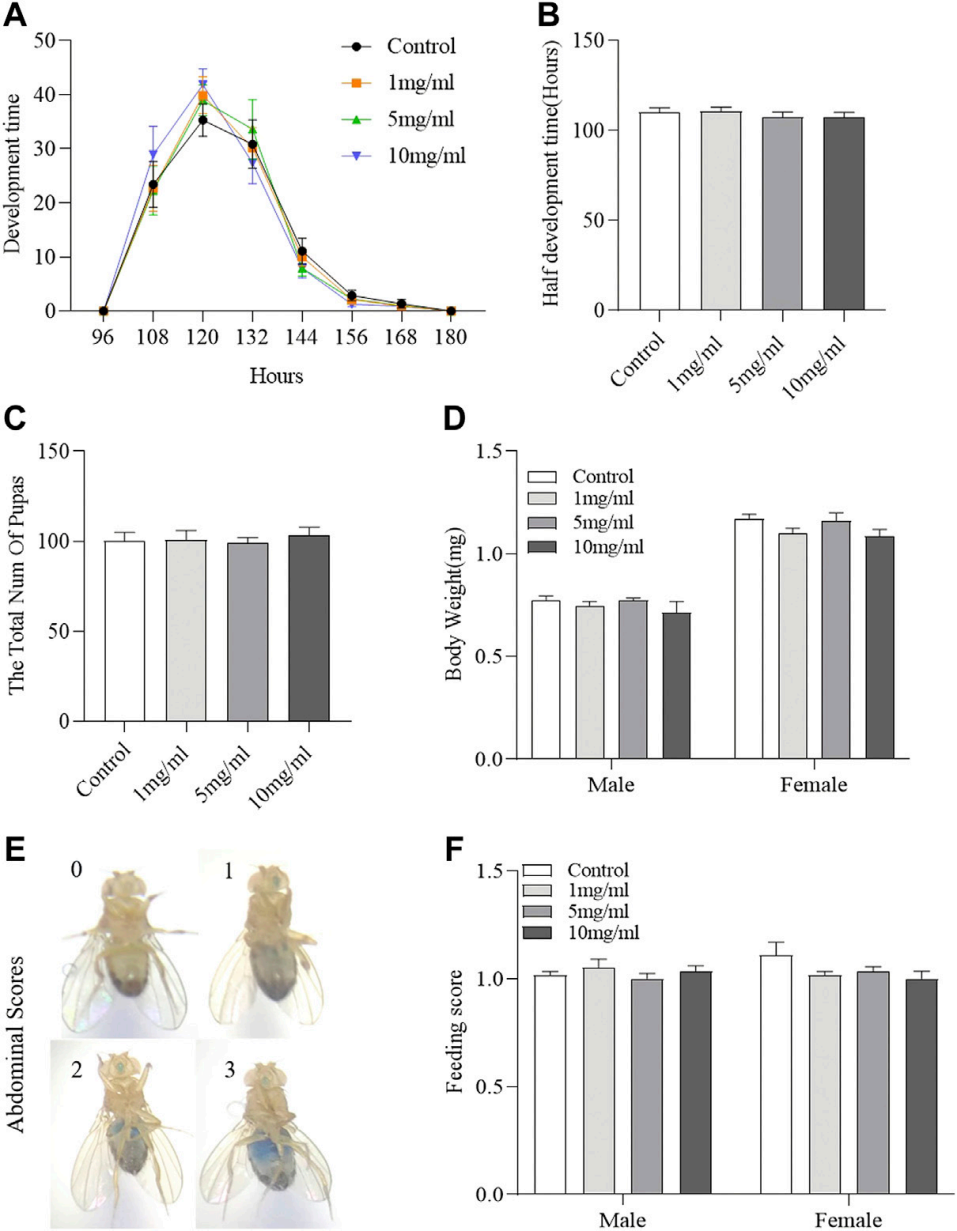
Daily intake of FPE does not affect development, body weight and food consumption. Flies were develop in standard foods (control groups), or foods supplemented with FPE (1, 5, 10 mg/L). During their development, pupation time **(A)**, half development time of flies **(B)** and the total number of pupas **(C)** are recorded (*n* = 20). After eclosion of the flies fed the food with or without FPE, the body weight (*n* = 10) **(D)** and feed consumption **(F)** of adult females and males is not affected (*n* = 56–60). **(E)** Representative examples of flies that consumed different amounts of food, visualized as blue dye. The results are presented as the means ± SEM.

*Drosophila* development is considered to be a high-throughput method for assessing drug safety (Abnoos et al., 2013; Gao et al., 2020). To determine the effect of FPE on development, we analyzed the effect of FPE on growth phase and hatchability. FPE supplementation did not regulate the growth phase from egg to pupa (Figures 3A,B) and also did not affect hatchability (Figure 3C). It indicates that FPE has no role to mediate development. In addition, the body weight was determined in adult flies after hatching (Figure 3D). Flies fed with FPE at 1, 5 and 10 mg/ml had the similar body weight with control flies. Next, we attempted to determine the possible effect of FPE (1, 5 and 10 mg/ml) on food intake (Figures 3E,F). As a control, the total amount of food consumption was not altered when flies fed with dose-dependent FPE. Thus, these results indicate that FPE has no effect on the development or metabolism in flies.

### Flos puerariae extract maintains intestinal morphological integrity following sodium dodecyl sulfate ingestion

**FIGURE 4:**
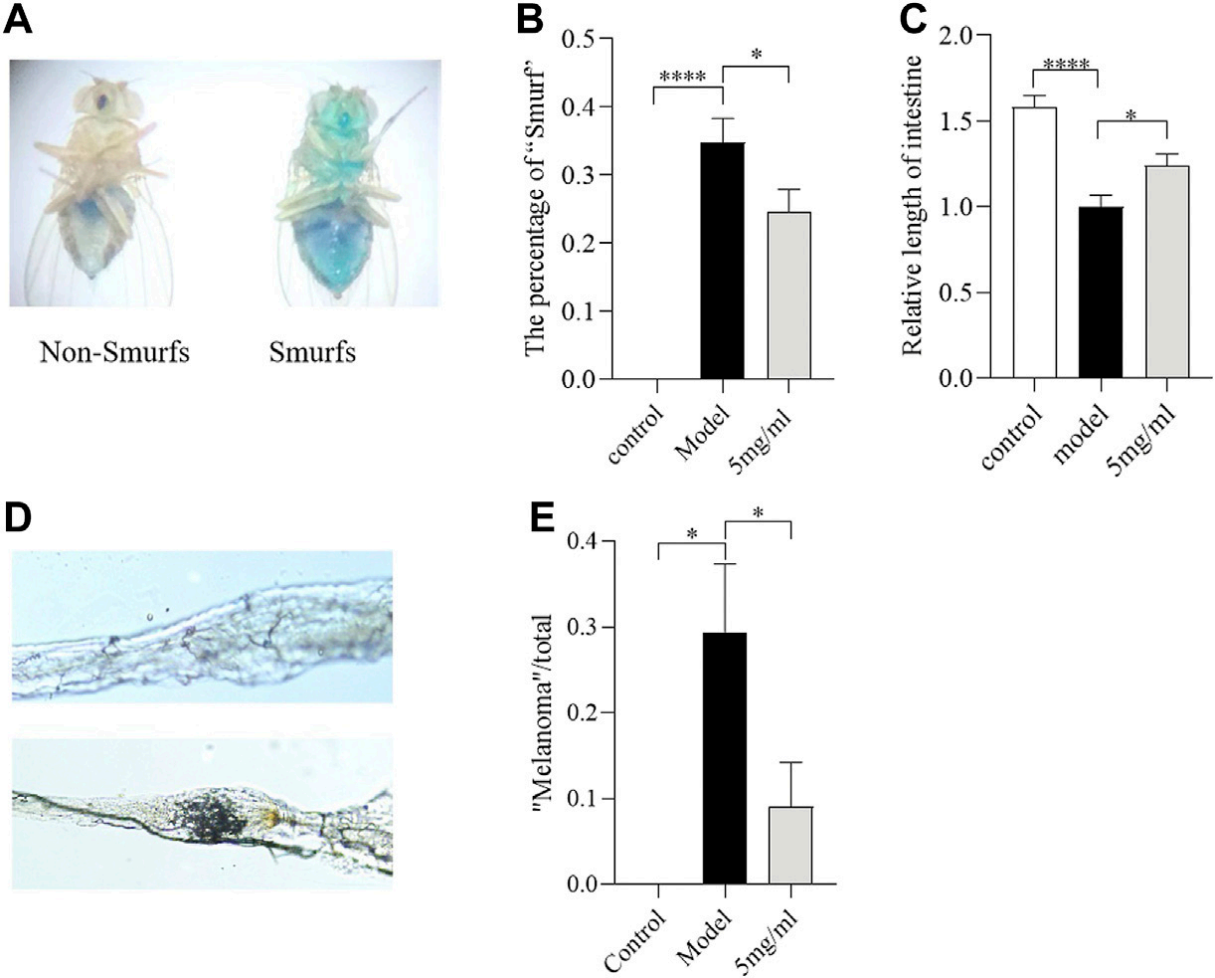
The FPE ameliorates the morphological change of the intestine against SDS-induced injury. **(A)** Schematic diagram of flies that underwent the Smurf assay. The percentage of “Smurf” (*n* = 170–190) **(B)** and relative length of intestine (*n* = 12) **(C)** in control flies or FPE treated flies that fed with 0.6% SDS. **(D)** Nomarski images of the fly gut. Up panel, “Non-malanotic mass”; down panel, “malanotic mass”. **(E)** The percentage of “malanotic mass” flies (*n* = 33–34). *p < 0.05, ****p < 0.001 vs. the model group.

The ingestion of SDS could induce melanotic tumors and morphological changes in the *Drosophila* intestine (Liu et al., 2016). To evaluate whether FPE maintains intestinal morphological integrity under SDS stimulation, the “Smurfs”, intestinal length and melanotic tumors were tested in females after 0.6% SDS treatment for 3 days. The “Smurfs” experiment was used to determine the integrity of the intestinal epithelial barrier (Rera et al., 2011). Approximately 35% of SDS-treated flies had “Smurfs” in the gut, and the proportion of “Smurfs” decreased to 25% after 5 mg/ml FPE treatment (Figures 4A,B). SDS supplementation significantly shorted the intestinal length (Figure 4C). Following SDS treatment, female flies fed with 5 mg/ml FPE had longer intestinal length than control flies. About 30% “melanotic tumors” were observed in SDS treated flies, and it was decreased to about 12% after flies fed with FPE (Figures 4D,E). Together, these results suggest that SDS ingestion disrupts the intestinal barrier and morphology, and FPE has protective function in flies.

### Flos puerariae extract inhibits the intestinal stem cells proliferation induced by sodium dodecyl sulfate in the gut

**FIGURE 5:**
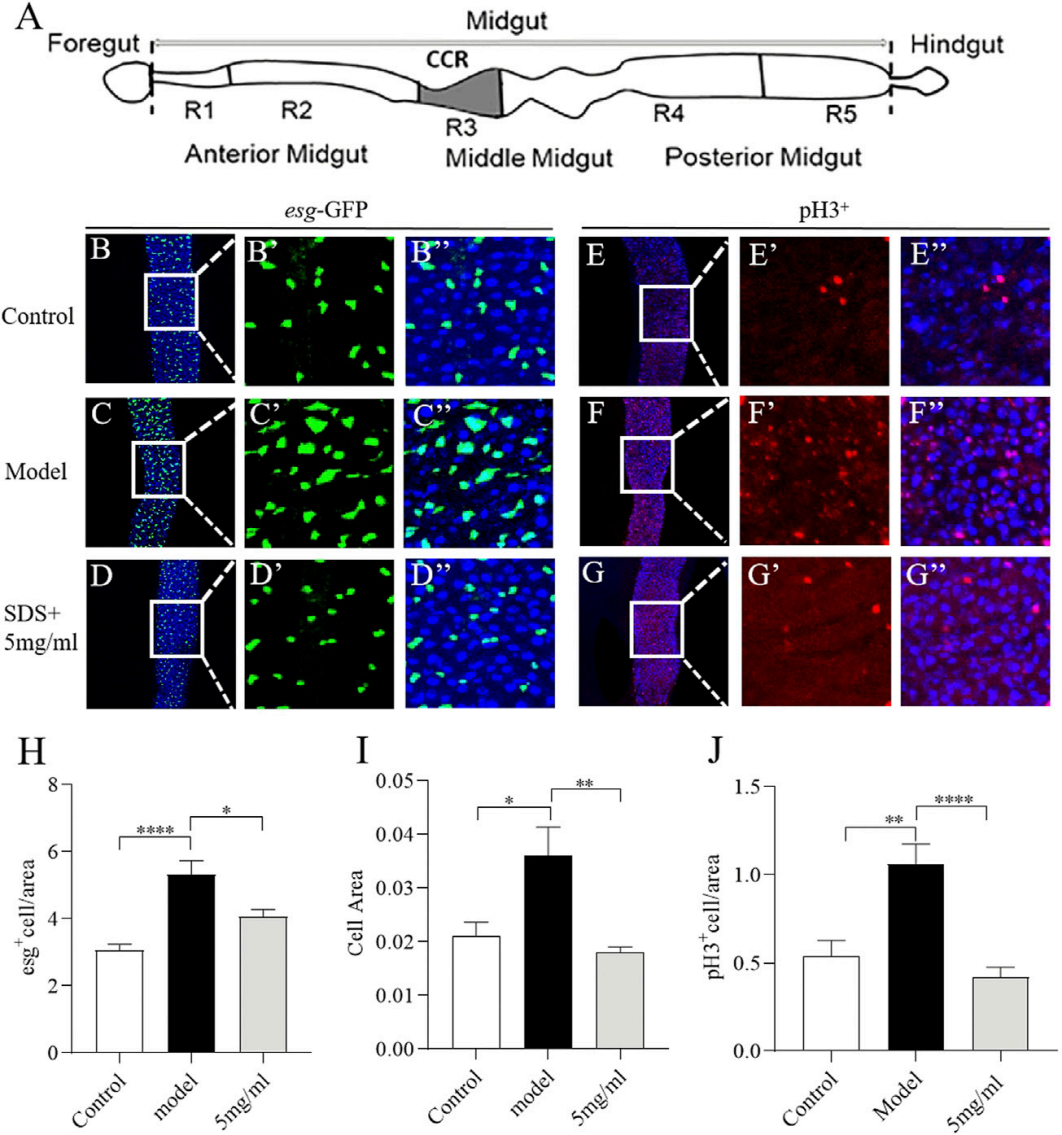
FPE protects intestinal stem cells (ISCs) from toxic chemical-induced proliferation and differentiation. **(A)** Schematic diagram of the fly gut, and the dissected site for immunostaining (grey). **(B–D)** Immunofluorescence images of the dissected midguts of *esg-*GFP in control flies and 0.6% SDS stimulated flies fed with or without 5 mg/ml FPE. *esg-*GFP: ISC/enteroblast cell marker (green). Quantification of the relative size **(H)** and number **(I)** of esg positive cells (*n* = 13–15). **(E–G)** Immunofluorescence images of the pH3 positive cells in control flies and 0.6% SDS stimulated flies fed with or without 5 mg/ml FPE. The guts of flies were labeled with anti-pH3 (Red) and DAPI (blue). **(J)** Quantification of the number of pH3 positive cells (*n* = 11–13). *p < 0.05, **p < 0.01, ****p < 0.0001 vs. the model group.

Intestinal morphology damage leads to the activation of intestinal stem cells (ISCs) proliferation to regenerate the damage intestinal epithelium (Wu et al., 2020). We next investigate the effect of FPE on ISCs proliferation induced by SDS (Figure 5A). First, we characterized the number of dividing cells using Escargot (Esg), a specific marker of stem cells and enteroblasts (Korzelius et al., 2014). Esg-Gal4; UAS-GFP reporter gene was only rarely expressed in a small, dispersed subset of rounded cells in the intestinal epithelium (Figure 5B). SDS stimulation led to the significant increase in the area and number of GFP-positive cells along the gut, which indicates an extensive increase in stem cell-derived cells (Figure 5C). 5 mg/ml FPE supplementation reduced the increased area and number of GFP-positive cells in the gut (Figures 5D–I). To extend this finding, we used an anti-phosphohistone H3 (anti-PH3) antibody that marks dividing cells to stain gut. A high number of PH3-positive cells were detected in the intestinal epithelium of SDS treated flies, while a very low number of PH3-positive cells in control flies gut (Figures 5E,F). 5 mg/ml FPE supplementation significantly decreased the number of PH3-positive cells in the gut (Figures 5D–J). Thus, FPE can inhibit the ISCs abnormal proliferation induced by SDS.

### Flos puerariae extract inhibits the oxidative damage induced by sodium dodecyl sulfate in the gut

**FIGURE 6:**
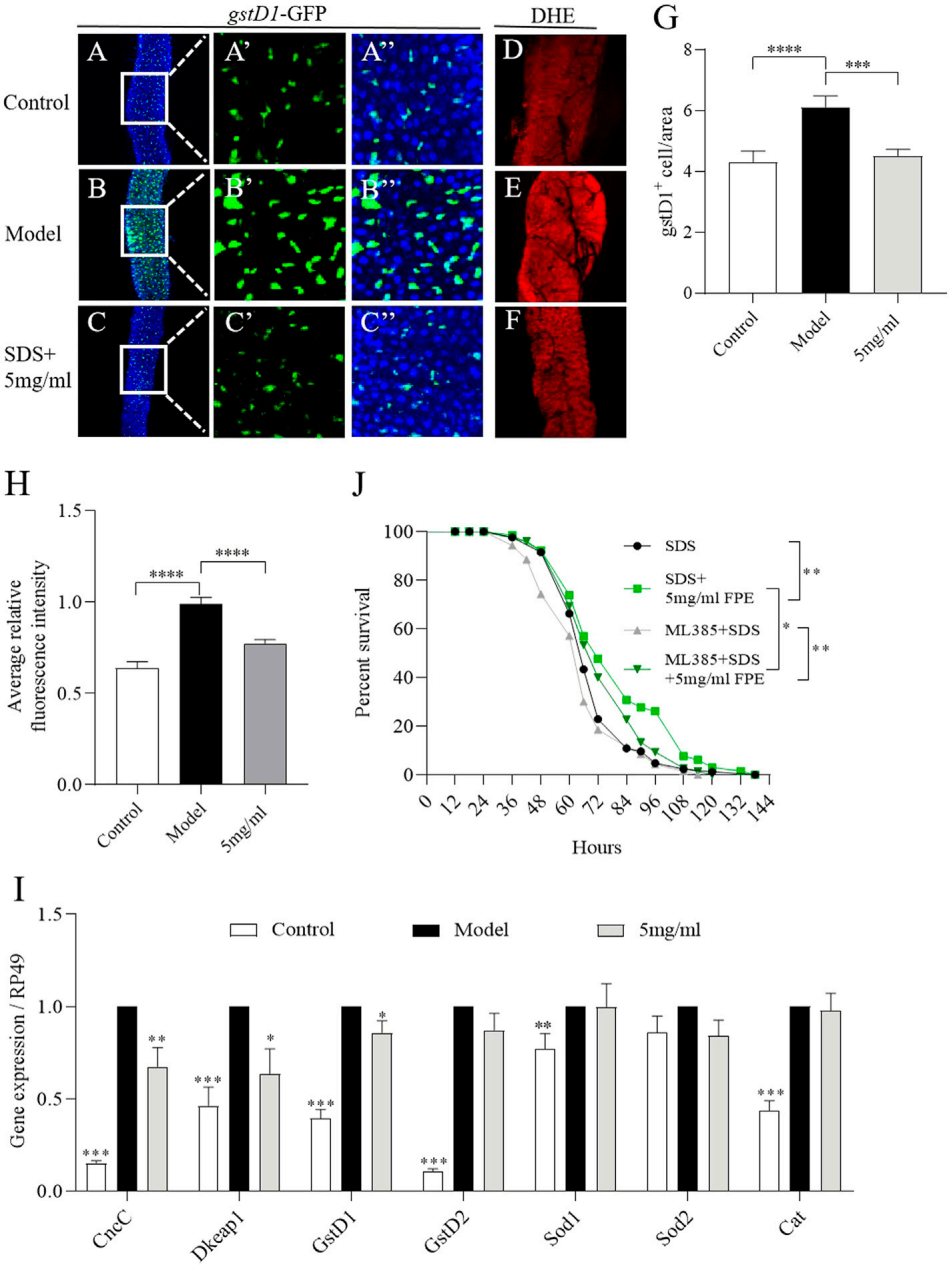
FPE decreases oxidative stress induced by SDS in the gut. **(A–C)** Immunostaining of the posterior midgut of adult *gstD1*-GFP flies that stimulated with or without 0.6% SDS for 16 h (*n* = 8–9). GFP staining highlighted the expression of *gstD1*-GFP (green). **(D–F)** ROS level was monitored using DHE. **(G)** Quantification of the number of *gstD1* positive cells in the midguts (*n* = 16–17). **(H)** Quantification of ROS level. **(J)** Survivorship curves in SDS treated four groups flies. **(I)** mRNA expression of *CncC*, *Keap1*, *gstD1*, *gstD2*, *Sod1* and *Cat* in the gut (*n* = 3). *p < 0 0.05, **p < 0.01, ***p < 0.001 vs. the model group (SDS-stimulated group).

Extensive ROS is produced in the gut under injury, and can stimulate ISCs proliferation and damage the intestinal epithelium (Zhang et al., 2020). To assess the effect of FPE on ROS levels during SDS stimulation, the *gstD1* (*gstD1-GFP*) transgenic flies sensitive to ROS were used (Zemanová et al., 2016; Tan et al., 2018). The activity of gstD1 is increased in differentiated cells and tissues of flies in response to stress (Tan et al., 2018). We determined the role of FPE on regulating ROS levels in intestinal epithelium under SDS stimulation. We found that under SDS stimulation, *gstD1-GFP* was expressed highly in intestinal epithelium (Figures 6A,B). 5 mg/ml FPE supplementation significantly inhibited the *gstD1-GFP* expression in SDS-treated flies (Figures 6C,G). Next we detected ROS level in the gut using dihydroethidium (DHE). After exposed to SDS for 72 h, flies had a robust intensity of fluorescence probe in the midgut compared to control flies (Figures 6D,E,H). 5 mg/ml FPE supplementation remarkably reversed the SDS-induced ROS accumulation (Figures 6F,H). These results suggest that FPE has function to protect the intestinal epithelium against SDS-induced oxidative damage.

To further certificate the role of FPE on regulating Nrf2/Keap1 signaling mediated by SDS, we detected the mRNA expression of *CncC*, *dKeap1*, *gstD1*, *gstD2*, *Sod1*, *Sod2* and *Cat* in gut. As depicted in Figure 6I, exposure of females to SDS for 16 h initiated the increased mRNA expression of *CncC*, *Keap1*, *gstD1*, *gstD2*, *Sod1* and *Cat.* After 5 mg/ml FPE treatment, *CncC*, *dKeap1* and *gstD1* expressions were recovered in SDS-treated flies (Figure 6I). To extend this studying, we fed Nrf2 inhibitor ML385 to inhibit the activity of Nrf2 in flies. 0.4 mm ML385 supplementation did not affect the survival rate under SDS stimulation in control flies, while it shorted the survival rate in FPE treated females (Figure 6J). 5 mg/ml FPE supplementation still extended the survival rate in ML385 treated females, following exposure to SDS. It indicates that FPE protect against SDS-induced injury via activating Nrf2/Keap1 signaling and other signaling.

### Flos Puerariae extract inhibits the activation of JAK-STAT pathway and Wnt pathway induced by sodium dodecyl sulfate in the gut

**FIGURE 7:**
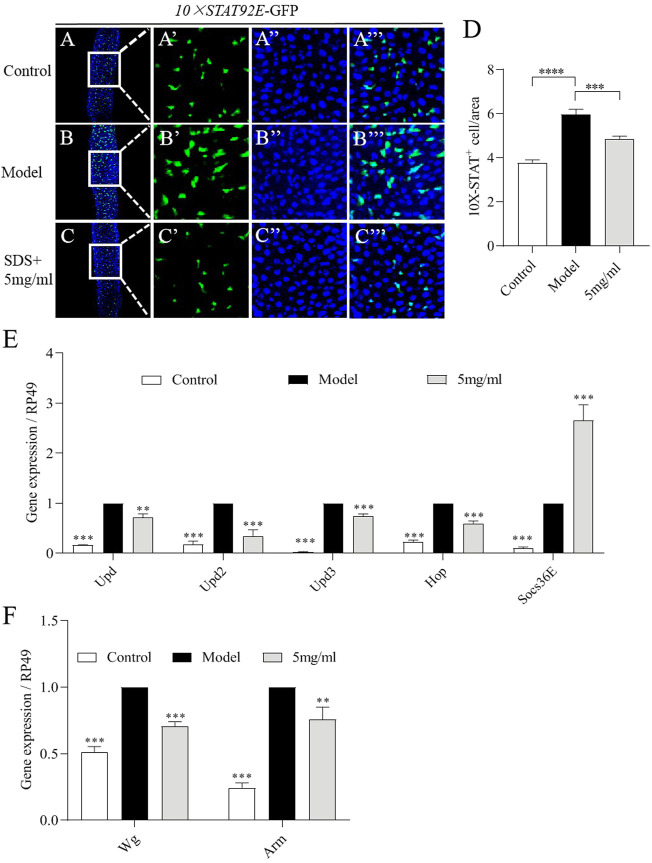
FPE restrains JAK-STAT signaling and Wnt signaling pathways in SDS-stimulated gut. **(A–C)** Immunostaining of adult female posterior midguts in *10×STAT92E-*GFP flies after 16 h treatment with or without 0.6% SDS. GFP staining highlighted *10×STAT92E-*GFP expression (green). **(D)** Quantification of the numbers of *10×STAT92E* positive cells in the midguts (*n* = 29). **(E)** mRNA levels of Upds (*Upd*, *Upd2* and *Upd3*) and JAK-STAT pathway (*Hop* and *Socs36E*) from midgut in control flies and 0.6% SDS stimulated flies fed with or without 5 mg/ml FPE (*n* = 3). **(F)** mRNA levels of Wnt signaling (*Wg* and *Arm*) in the gut (*n* = 3).*p < 0 0.05, **p < 0.01, ***p < 0.001 vs. the model group (SDS- stimulated group).

To further examine the molecular mechanism of FPE on protecting the intestinal injury, firstly network pharmacology analysis was used. JAK-STAT signaling pathway were defined as one of core pathways (Supplementary Figure S1). The JAK-STAT pathway can be activated by cytokines, such as the Unpaired family (UPD, UPD2 and UPD3) in the midgut (Zhou et al., 2013). We then investigated the activity of JAK-STAT pathway in the gut of flies. A fly line carrying a reporter construct composed of ten tandem Stat92E DNA binding sites inserted upstream of a minimal promoter and a *GFP* reporter (referred to as *10×STAT92E-GFP*) (Bach et al., 2007). We found a weak GFP signal was present in intestinal epithelium of control flies (Figure 7A). The increased number of GFP-positive cells was detected in the gut after SDS stimulation for 16 h (Figure 7B). However, 5 mg/ml FPE supplementation remarkably inhibited the increased levels of GFP expression along the gut in SDS stimulated flies (Figures 7C–D). It indicates that SDS can activate JAK-STAT pathway in the gut, which is inhibited by FPE. Then, we monitored the mRNA levels of Upds (*Upd*, *Upd2* and *Upd3*) and JAK-STAT pathway (*Hop* and *Socs36E*) (Bach et al., 2007). SDS stimulation increased expression of *Upd*, *Upd2, Upd3, Hop* and *Socs36E* in the gut (Figure 7E). mRNA expression levels of *Upd*, *Upd2*, *Upd3* and *Hop* was decreased, but *Socs36E* expression was increased in SDS stimulated flies fed with 5 mg/ml FPE compared to flies without FPE treatment (Figure 7E). It indicates that FPE has function to inhibit the increased JAK-STAT pathway induced by SDS in the gut. The activation of Wnt signaling pathway is a classic feature of chronic IBD and its animal models (Bradford et al., 2017; Lee et al., 2021). We measured mRNA levels of *Wg*, the homologue of wnt-1, and *Arm* as a homology of β-catenin in the gut (Clevers, 2006). SDS ingestion resulted in a significant increase in *Wg* and *Arm* gene expression in the intestine, which was reduced by FPE intervention (Figure 7F). It suggests that FPE can inhibit the activation of the Wnt signaling pathway. Together, these results indicate that FPE has function to protect intestinal homeostasis and ISCs proliferation by inhibiting JAK-STAT pathway and Wnt signaling pathway.

## Discussion

**FIGURE 8:**
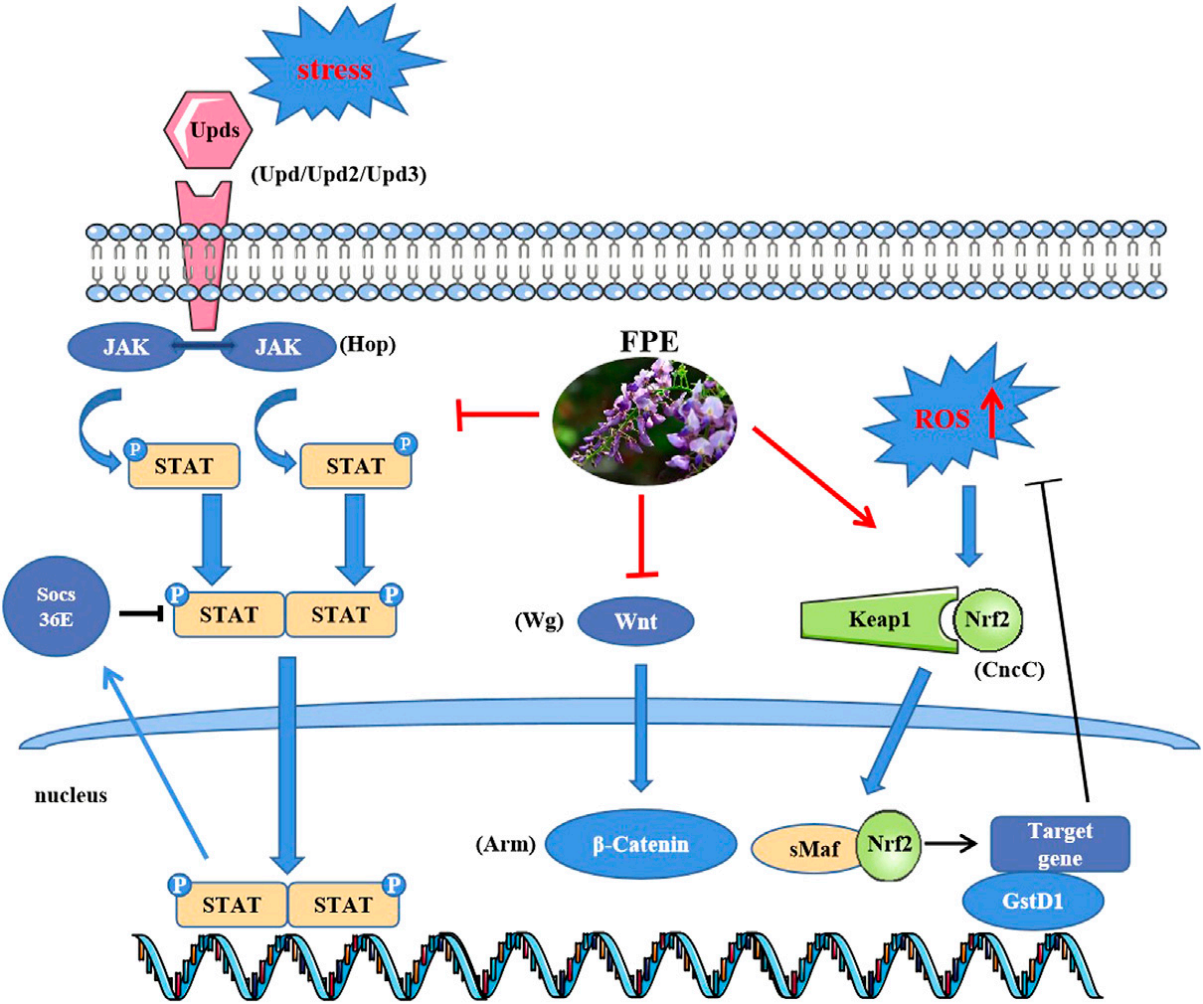
Functional mechanism of FPE in the treatment of IBD.

The intestinal epithelium is a prominent defense barrier against invasion and facilitates host-microorganism interactions (Nagai et al., 2021), and disruption of intestinal integrity leads to several intestinal disorders. Here, we dissected the role of FPE in protecting against SDS-induced intestinal injury. The results revealed that the FPE supplement maintained intestinal homeostasis and protected the integrity of the intestinal barrier mainly by regulating Nrf2-Keap1, JAK-STAT and Wnt signaling pathways (Figure 8).

*Drosophila* development is considered to be a high-throughput method for assessing drug safety (Legaz et al., 2016; Gao et al., 2020). We found FPE supplementation had no effect on hatching rates and growth rates during development in flies. It indicates FPE does not promote development and has no toxic effect. In addition, flies fed with FPE during development had no function to regulate body weight and food intake compared to control flies, implying that FPE does not regulate metabolism. However, some studies reported that FPE supplementation effectively inhibited metabolic disease-induced body weight gain and fat accumulation in mice, which was not quite consistent with our results (Kubo et al., 2012; Satpathy et al., 2021). That probably because we discuss the effect of FPE on the normal state metabolism of the organism, and find the daily diet is not harmful to the organism.

Previous studies have shown that toxic chemicals lead to IBD in animals, various Chinese medicine can treat IBD by improving the survival rate and alleviating the colonic shortening (Rodriguez-Canales et al., 2020). Our results showed that administration of FPE reduced the severity of SDS induced colitis; this statement was supported by survival rate that FPE substantially enhanced the resistance against SDS- derived constant stressors. In addition to the survival rate, intestinal functions, such as intestinal barrier integrity, digestive function, etc., are closely related to intestinal homeostasis during inflammation (Sommer et al., 2017; Wang et al., 2021). Intestinal barrier dysfunction accompanied by increased intestinal permeability is one of the typical symptoms in the pathophysiology of IBD (Hofma et al., 2018). The ingestion of SDS induces melanotic tumors and morphological changes in the *Drosophila* intestine (Song et al., 2021). We found that FPE significantly protected intestinal barrier integrity, inhibited colonic shortening and decreased the percentage of melanotic tumors in SDS treated flies. Previous studies showed that FPE supplementation significantly prevent LPS induced inflammatory stress and oxidative stress in LPS-induced inflammatory model (Lertpatipanpong et al., 2020). These findings indicate that FPE has a role to protect the intestinal barrier and integrity in inflammatory conditions. Furthermore, since ingestion of SDS could cause systemic injury beyond the intestine, we could not rule out the possibility that FPE supplementation might also be beneficial for other tissues and cells, thereby increasing the survival of flies in response to SDS stimulation. Further work is required to identify a possible protective role of FPE in other tissues, following SDS stimulation *in vivo*.

Ingestion of toxic chemicals and intestinal infections with bacteria could lead to intestinal cell damage and disrupt the homeostasis of the intestinal epithelium (Wu et al., 2020; Song et al., 2021). In SDS-stimulated flies, the ISCs undergo an increase in proliferation and a decrease in differentiation, which promotes a significant rise of the intestinal stem and progenitor cells number (Wu et al., 2020). We found FPE supplementation inhibited SDS- induced ISC hyperproliferation. Thus, FPE supplementation may promote intestinal homeostasis and ISC health against intestine damage. ROS plays an important role in maintaining intestinal homeostasis in organisms (Bhattacharyya et al., 2014). Excessive ROS induces oxidative stress, resulting in intestinal disorder and ISCs over-proliferation (Wisidagama & Thummel, 2019; Zhang et al., 2020). We found that FPE could inhibit the accumulation of ROS induced by SDS in the midgut. Nrf2 plays a crucial role in cellular detoxification in response to oxidative stress through regulation of antioxidant enzymes and proinflammatory cytokines (Liu et al., 2021; Piotrowska et al., 2021). Nrf2 inhibitor ML385 decreased the extended the survival rate induced by FPE in SDS treated flies, without abolished this phenotype. It indicates that FPE protects SDS-induced injury via activating the Nrf2/Keap1 signaling pathway.

Activation of the JAK-STAT and Wnt signaling pathways are associated with gastrointestinal inflammation and ISCs proliferation, which may accelerate the development of IBD (Coskun et al., 2013; Piotrowska et al., 2021). In *Drosophila*, the JAK-STAT and Wnt signaling pathways are highly correlated with intestinal homeostasis during acute stress or injury (Wang et al., 2019b; Kim et al., 2019). Toxic compounds induced the epithelial cell damage by releasing of cytokines (*Upd, Upd2*, *Upd3*) to activate JAK-STAT pathway in the gut of flies (Chakrabarti et al., 2016; Song et al., 2021). In this study, FPE supplementation remarkably inhibited the increased levels of GFP expression that targeted STAT92E along the gut in SDS stimulated flies. Further RT-qPCR analysis showed that SDS promoted the expression levels of JAK-STAT related genes *Upd*, *Upd2*, *Upd3*, *Hop* and *socs36E* in the gut, while administration of FPE reduced the expression of these genes and increased the expression of *socs36E*. Upd, Upd2 and Upd3 are the major cytokine and ligand for the JAK-STAT signaling pathway in *Drosophila* and activate the JAK-STAT signaling pathway during stress (Zhou et al., 2013), while the activity of the JAK-STAT signaling pathway was down regulated by *socs36E*, a suppressor of cytokine signaling (SOCS) protein (Callus & Mathey-Prevot, 2002). Thus, FPE decreased JAK-STAT signaling pathway possibility by decreasing the inflammatory cytokines. Ingestion of toxic chemicals leads to increased expression of *Wg* in the intestine and activation of the Wnt signaling pathway (Lee et al., 2021). In contrast, the gene expressions of *Wg* and *Arm* were down-regulated after FPE intake. Together, FPE maintain intestinal homeostasis in inflammatory environment via inhibiting the JAK-STAT and Wnt signaling pathways, and activating Nrf2/keap1 signaling pathway. It is unknown whether there are other pathways that participate in this process. Network pharmacology analysis showed that many targets were enriched in inflammation-related signaling pathways, such as JAK-STAT signaling pathway, NF-kappa B signaling pathway, PI3K-Akt signaling pathway and Toll-like receptor signaling pathway (Supplementary Figure S1). Dorsal (Drs) and Dorsal-related immunity factor (Dif) as the NF-kappa B factors could be activated by Myeloid differentiation factor 88 (Myd88) in flies (Zhang et al., 2012a). Our RT-PCR results showed that FPE decreased the enhanced mRNA levels of *Drs*, *Dif* and *Myd88* induced by SDS in the *Drosophila* gut (Supplementary Figure S2), which means FPE could inhibit NF-kappa B signaling pathway. Additional protective pathways activated by FPE need to be further explored in the future.

Pueraria lobata is an abundant source of isoflavones (Yao et al., 2021). Here, six active isoflavones were examined and identified as puerarin, daidzein, daidzin, genistein, rutin and apigenin in FPE using LC-MS techniques, which is consistent with previous studies (Liu et al., 2012; Thapa et al., 2022). Network pharmacology analysis of the active components of Pueraria lobata for the treatment of IBD revealed that the biological activity of Pueraria lobata was also mainly reflected in isoflavones (Supplementary Table S1). Studies have shown that STAT3 and Keap1 are key targets for the treatment of IBD, so we used as targets for molecular docking (Lu et al., 2016; Kasembeli et al., 2018) (Supplementary Table S2). Results indicated that rutin and daidzin has a good docking affinity with STAT3 (Supplementary Figure S3A,B). Li found that constituents in Sophorae Flos, such as rutin, quercetin and luteolin, can inhibit STAT3 signaling to reduced cell viability (Li et al., 2017). Yao and colleagues found daidzin significantly reduced the expression of inflammatory genes and cell proliferation signaling molecules via JAK2/STAT3 (Yao et al., 2021). Molecular docking showed puerarin and daidzin had good docking affinity with Keap1 (Supplementary Figure S3C,D). Therefore, we speculate that puerarin, daidzin and rutin in FPE may be key components in the treatment of IBD, which still needs to be further elucidated.

Collectively, our study demonstrated that FPE displayed clear protective effects, including anti-inflammatory and antioxidant effects, and the modulation of intestinal epithelial barrier in SDS-induced injury. Further studies revealed that FPE could remarkably attenuate the SDS-induced excessive ISC proliferation and differentiation. Moreover, the protective effect of FPE on IBD is mainly mediated by the Nrf2/keap1, JAK-STAT and Wnt signaling pathways. Therefore, our results provided evidence for FPE as a potential alternative functional foods and medicines for the prevention and treatment of IBD.

## Data Availability

The datasets presented in this study can be found in online repositories. The names of the repository/repositories and accession number(s) can be found in the article/Supplementary Material.
