# Supplementary figures and images for: Flos puerariae ameliorates the intestinal inflammation of Drosophila via modulating the Nrf2/Keap1, JAK-STAT and Wnt signaling
Source: Front Pharmacol. 2022 Aug 17;13:893758. doi: 10.3389/fphar.2022.893758 (PMC9432424; doi:10.3389/fphar.2022.893758)

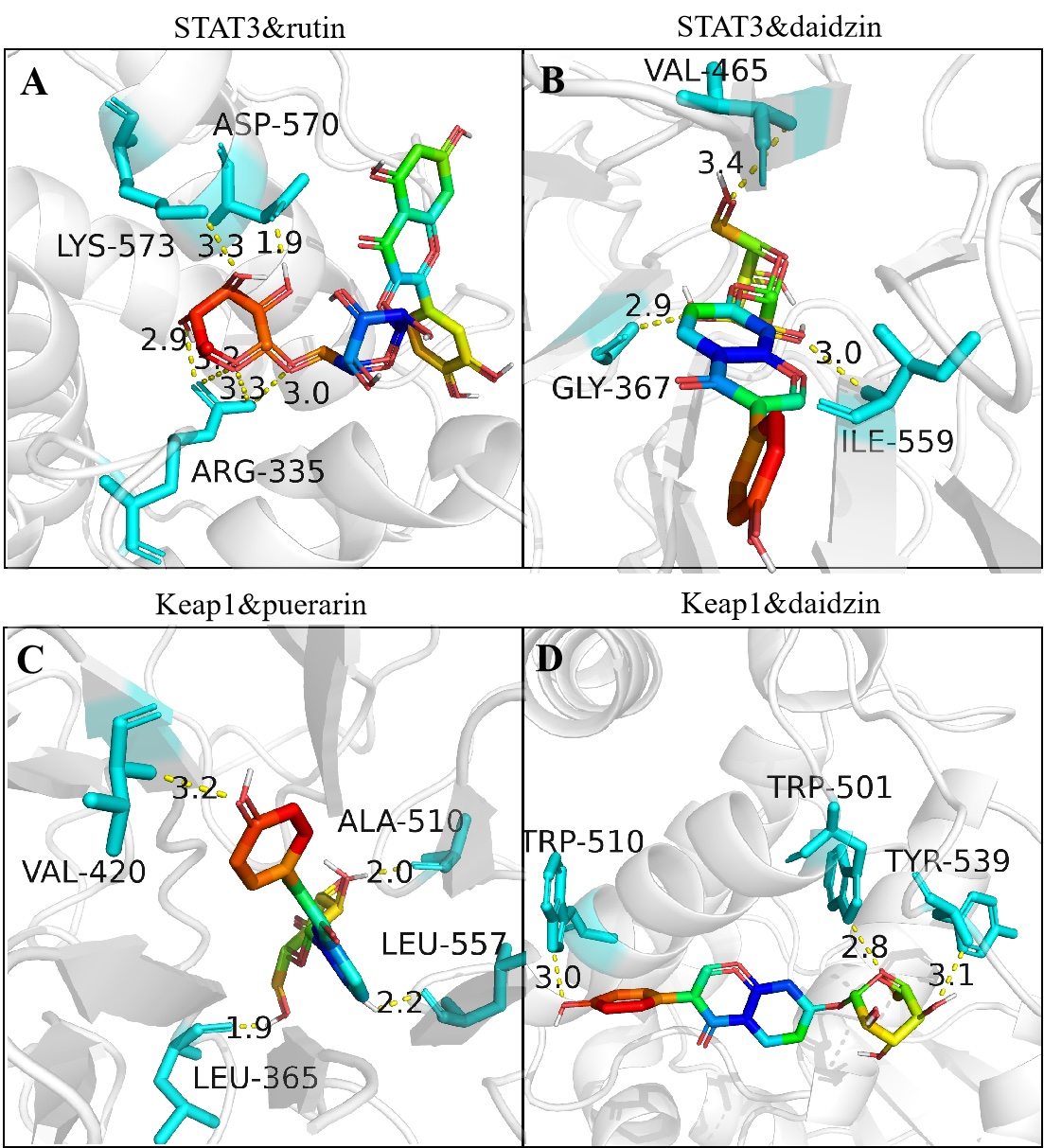

Supplement: Supplementary file 1 [file Image3.JPEG]

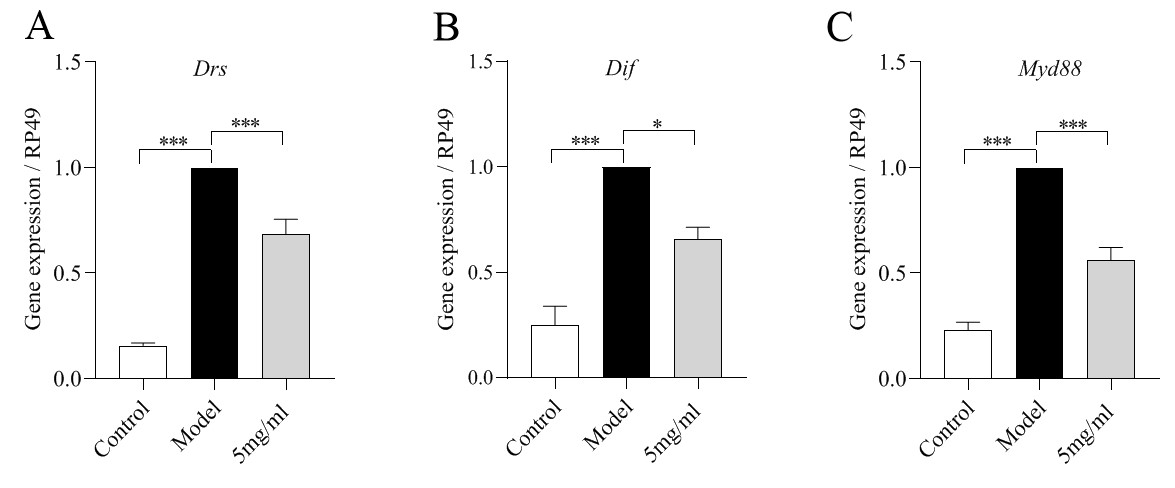

Supplement: Supplementary file 2 [file Image2.JPEG]

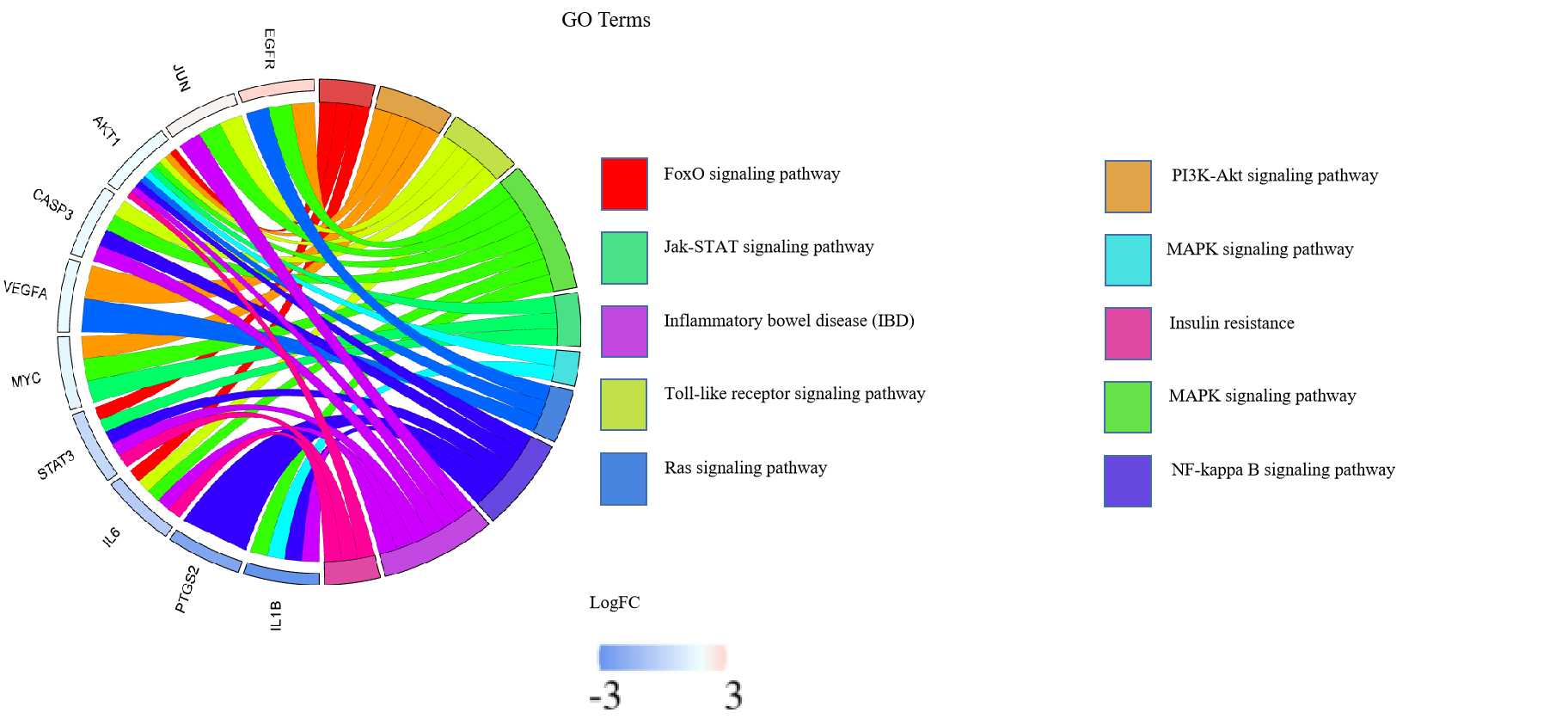

Supplement: Supplementary file 3 [file Image1.TIF]
