# Supplementary material for: Flos puerariae ameliorates the intestinal inflammation of Drosophila via modulating the Nrf2/Keap1, JAK-STAT and Wnt signaling
Source: Front Pharmacol. 2022 Aug 17;13:893758. doi: 10.3389/fphar.2022.893758 (PMC9432424; doi:10.3389/fphar.2022.893758)
Supplement: Supplementary file 4 [file DataSheet1.docx]

| Mol ID | Molecule Name | OB (%) | DL |
| --- | --- | --- | --- |
| MOL000098 | quercetin | 46.43 | 0.28 |
| MOL000358 | beta-sitosterol | 36.91 | 0.75 |
| MOL000359 | sitosterol | 36.91 | 0.75 |
| MOL000392 | formononetin | 69.67 | 0.21 |
| MOL000422 | kaempferol | 41.88 | 0.24 |
| MOL000449 | Stigmasterol | 43.83 | 0.76 |
| MOL000468 | 8-o-Methylreyusi | 70.32 | 0.27 |
| MOL001749 | ZINC03860434 | 43.59 | 0.35 |
| MOL001792 | DFV | 32.76 | 0.18 |
| MOL002959 | 3'-Methoxydaidzein | 48.57 | 0.24 |
| MOL003629 | Daidzein-4,7-diglucoside | 47.27 | 0.67 |
| MOL004957 | HMO | 38.37 | 0.21 |
| MOL005916 | irisolidone | 37.78 | 0.3 |
| MOL008400 | glycitein | 50.48 | 0.24 |
| MOL011791 | Kakkalide | 46.91 | 0.67 |
| MOL011793 | kakkatin | 55.25 | 0.24 |
| MOL012976 | coumestrol | 32.49 | 0.34 |
| MOL013305 | Garbanzol | 83.67 | 0.21 |
| MOL000390 | daidzein | 19.44 | 0.19 |
| MOL000415 | rutin | 3.2 | 0.68 |
| MOL000481 | genistein | 17.93 | 0.21 |
| MOL009720 | daidzin | 14.32 | 0.73 |
| MOL012297 | puerarin | 24.03 | 0.69 |

Table S1 The active ingredients of Flos Puerariae

Table S2 The binding energy of Active ingredient

| Active ingredient | Keap1 (6TYM) docking  score (kcal/mol) | STAT3 (6NU) docking  score (kcal/mol) |
| --- | --- | --- |
| puerarin | -10.5 | -6.6 |
| daidzin | -10.3 | -7.3 |
| rutin | -9.4 | -8.0 |
| genistein | -9.0 | -6.4 |
| apigenin | -9.2 | -7.0 |
| daidzein | -8.4 | -6.3 |
